# Sentinel Animals in a One Health Approach to Harmful Cyanobacterial and Algal Blooms

**DOI:** 10.3390/vetsci3020008

**Published:** 2016-04-21

**Authors:** Lorraine C. Backer, Melissa Miller

**Affiliations:** 1 National Center for Environmental Health, Centers for Disease Control and Prevention, 4770 Buford Highway NE, MS F-60, Chamblee, GA 30341, USA; 2 Office of Spill Prevention and Response, Department of Fish and Wildlife, Marine Wildlife Veterinary Care and Research Center, 1451 Shaffer Rd, Santa Cruz, CA 95060, USA; melissa.miller@wildlife.ca.gov; 3 School of Veterinary Medicine, University of California at Davis, Davis, CA 95616, USA

**Keywords:** algae, biotoxin, cyanobacteria, cyanotoxin, domoic acid, freshwater, harmful algal bloom, marine water, one health, saxitoxin, sentinel

## Abstract

People, domestic animals, and wildlife are all exposed to numerous environmental threats, including harmful algal blooms (HABs). However, because animals exhibit wide variations in diet, land use and biology, they are often more frequently or heavily exposed to HAB toxins than are people occupying the same habitat, making them sentinels for human exposures. Historically, we have taken advantage of unique physiological characteristics of animals, such as the sensitivity of canaries to carbon monoxide, to more quickly recognize threats and help protect human health. As HAB events become more severe and widespread worldwide, exposure and health outcome data for animals can be extremely helpful to predict, prevent, and evaluate human exposures and health outcomes. Applying a One Health approach to investigation of HABs means that lessons learned from animal sentinels can be applied to protect people, animals and our shared environment.

## 1. Introduction

Like humans, domestic animals and wildlife can experience acute and chronic health impacts following exposure to environmental threats, such as air or water pollution. Because of their unique habitat use, behavior, and biology, animals may be exposed to environmental pollutants more rapidly and at higher concentrations than humans residing in the same areas, making them potential sentinels for emerging threats to human health. Animal-based sentinel systems can expedite recognition of dangerous environmental conditions, illustrate pollutant bioavailability, and clarify health hazards associated with known contaminants, providing an approximation of what people might experience [1]. Historically, animal sentinels have been used *in situ* to monitor potentially dangerous environments. One well-known example is the use of canaries and mice in coal mines to warn miners when carbon monoxide was present at dangerous levels [2]. Animals live alongside humans in diverse environments, including homes, workplaces and natural aquatic or terrestrial ecosystems, and study of their pollutant exposure trends can provide early warnings of potential human health hazards [1].

As one important example, investigation of deaths of domestic animals and wildlife has expedited recognition of human health threats associated with exposure to harmful cyanobacterial and algal blooms (herein referred to as HABs) in water and food worldwide. The first published description of animals as sentinels for a dangerous HAB event was a short report by Francis [3] describing poisonings of domestic animals that drank from lakes of the Murray River estuary system in Australia. A severe cyanobacterial bloom was tentatively identified as *Nodularia spumigera*, and horses, sheep, dogs, and pigs died within hours of drinking from bloom-contaminated waters. Schwimmer and Schwimmer [4] reviewed historical reports of domestic and wild animal poisonings during freshwater HAB events, and Stewart *et al.* [5] published an updated review as part of a compendium on the current state of knowledge about cyanobacteria and future research needs.

Synergizing efforts to monitor sentinel animal events and sentinel animal systems with human disease surveillance activities is a critical component of the “One Health” approach for identifying and addressing environmental threats. One Health is a global initiative to link human, animal, and environmental health [6]. One Health principles encompass data integration, collaboration, and cooperation across all health disciplines, synergizing the study of human and animal health and disease in shared habitats. One of the greatest challenges is facilitating and expediting communication across human and animal health disciplines. HABs affect every aspect of global aquatic environments and thus represent a unique opportunity and an obvious need for interdisciplinary collaboration to identify, mitigate, and prevent adverse health outcomes. In this paper we review examples of sentinel animal events that have contributed to our collective understanding of potential public health threats from HABs, and discuss opportunities whereby investigation of HAB events using the One Health approach can expedite, optimize and guide current and future public health protections.

## 2. Example Animal Sentinel Systems and Events

### 2.1. Aquatic Species

Aquatic animals, including invertebrates, fish and marine animals, provide some of the earliest alerts regarding potential adverse effects from HABs, and face numerous health and survival threats from toxin exposure.

#### 2.1.1. Invertebrates

Marine and freshwater invertebrates, including mussels, clams and crabs, comprise an important food resource for animals and people living in both coastal and inland communities. Invertebrate population health reflects the quality of local marine waters and adverse changes may signal subsequent health risks for the people and animals that consume them or otherwise share the local environment. Invertebrates have served as sentinels for coastal and freshwater environmental contamination for at least 30 years. One example is the Mussel Watch program, the longest-running chemical contaminant monitoring program in U.S. coastal and Great Lakes Waters [7]. This program has monitored over 150 organic and inorganic contaminant concentrations in water and bivalves since 1986, including trends for polyaromatic hydrocarbons and polychlorinated biphenyls in sediments and bivalve tissues [8].

In addition to these legacy contaminants, the program has evolved to measure emerging contaminants of concern, including polybrominated biphenyl ethers (flame retardants) and pharmaceuticals. Mussel Watch data can be used to assess how increased regulation of these chemicals affects their environmental distributions and concentrations, and can help assess human health risks from consuming invertebrates, fish, or drinking water, or via recreational water contact. Archived specimens allow retrospective assessment of contaminants of concern, including new compounds and existing chemicals with newly discovered environmental distributions.

Over the past 20 years environmental monitoring programs have been expanded to include freshwater and marine HABs and their associated toxins. Like Mussel Watch, the sentinel animal event for these programs is the detection of toxins at concentrations of concern in shellfish. These programs monitor blooms and shellfish quality along coastlines where toxin-producing blooms occur, and provide early warnings of shellfish contamination specifically to protect human health (e.g., [9]). For example, Florida prevents most cases of neurotoxic shellfish poisoning through monitoring shellfish for brevetoxin, and posting harvest bans when levels are high [10].

In the Northeast, new technologies detect *Alexandrium fundyense,* which can develop into HABs often called “red tides”. During bloom events, dinoflagellates like *A. fundyense* can produce large concentrations of a potent neurotoxin (saxitoxin) that can bioaccumulate in shellfish and cause paralytic shellfish poisoning in animals and people. Models used data from ocean observing sensors to predict the concentrations of Alexandrium produce seasonal forecasts that support decisions to open or close commercial and sport shellfisheries for human consumption in the Gulf of Maine [11]. Data from these bivalve monitoring programs provide an evolving picture of coastal contaminants that can be used to assess risk for regional human exposure, to inform epidemiologic studies of exposures and health effects, and to optimize regional prevention, education, and outreach programs.

*Dinoflagellate* HAB events with abundant saxitoxin production are also an annual occurrence along the Pacific Coast of North America, and were the main catalyst for developing regional shellfish-based HAB monitoring programs. The Olympic Region Harmful Algal Bloom (ORHAB) partnership identified sampling sites along the coast and monitors for rapid increases in *Pseudo-nitzschia* cells and for the presence of toxin in seawater [12,13]. Using data from this monitoring program, resource managers can predict when domoic acid levels will be high in local shellfish and can target which recreational shellfish harvesting areas should be closed to prevent human poisonings. During the summer 2015, the largest and most extensive *Pseudo-nitzschia* bloom event ever recorded was documented off the Pacific Coast, extending from California to Alaska [14]. *Pseudo-nitzschia* produces domoic acid, and routine monitoring of ocean water and marine organisms detected elevated toxin levels beginning in May 2015. High domoic acid concentrations were documented in marine bivalves and crabs, resulting in extensive shellfish harvest closures [15]. High domoic acid levels in shellfish from these areas and could be an underlying cause of reported marine mammal deaths [16].

A common HAB event in Southeastern North America is Florida red tide, comprising seasonal blooms of the dinoflagellate *Karenia brevis* in the Gulf of Mexico. *Karenia brevis* produces the neurotoxin brevetoxin, which accumulates in the marine food web (e.g., [17]). Ongoing monitoring of shellfish and water by the Florida Fish and Wildlife Conservation Commission supports the management of commercial and recreational shellfish harvesting [18]. Shellfish beds are closed when *Karenia brevis* cells in water exceed 5000 per L, or when shellfish meat contains ≥20 mouse units of toxin [19]. Because of this diligence, very few cases of human intoxication from brevetoxins occur in Florida, and those typically involve visitors who may not understand or heed posted warnings. Florida provides public information on red tides, including when and where Florida red tide events occur and the status of shellfish harvesting beds, on the website of the Florida Fish and Wildlife Conservation Commission [20].

Finally, filter-feeding estuarine and marine invertebrates are a potential source of human and animal exposure to freshwater cyanotoxins originating in coastal rivers [21,22,23,24,25]. For example, filter-feeding invertebrates from regions of microcystin-contaminated coastal freshwater outflow are the most likely source of microcystin intoxication for threatened southern sea otters (*Enhydra lutris nereis*) in Monterey Bay, California [21]. As part of their investigation, Miller *et al.* (2010) [21] conducted laboratory studies showing that marine shellfish maintained under ambient conditions following exposure to cyanobacterial scum from a Pinto Lake bloom rapidly accumulate microcystins and only slowly depurate them, even when maintained in clean, uncontaminated seawater. Significant bioconcentration of microcystin by marine bivalves (clams, mussels and oysters) and snails, but not large marine crabs, was documented, with tissue concentrations of microcystin-LR up to 107 times higher in invertebrate hepatopancreas than in adjacent seawater. Findings from this investigation revealed a new environmental health threat for sea otters and people by tracing marine contamination by microcystins derived from fresh water, and demonstrating that marine shellfish consumed by both marine mammals and people can accumulate microcystins.

Microcystins have also been detected in wild and commercially-raised marine and estuarine mussels (*Mytilus trossulus*) from Puget Sound, Washington, D.C., U.S.; Nile tilpaia (*Oreochromis nitoticus*) from Chian Rai, Thailand; and other aquatic foods intended for human consumption [26,27,28]. Routine seawater and aquatic animal monitoring in areas where cyanotoxins have been found could provide early that warning that animal and human shellfish consumers could be at risk. However, the accuracy of detection requires further study, especially when attempting microcystin detection from biological samples [29]. Analytic detection of microcystins is complicated by the large diversity of microcystin congeners, challenges with congener extraction from biological samples, and a large range of abiotic and biotic processes that can alter the toxicity and structure of microcystin molecules [30]. When possible the most sensitive and specific sample extraction and detection methods should be used to screen biological samples, such as liquid chromatography-mass spectrometry (LC-MS), or liquid chromatography tandem mass spectrometry (LC-MS/MS).

The above summaries provide a snapshot of key HAB events that occur repeatedly along North American coastlines where invertebrates are exposed to HABs. Monitoring programs for these invertebrates can serve as sentinels for human and animal exposure, in addition to preventing foodborne exposures. Additional bloom events occur that are not captured in this overview. For example, the Florida coastline also experiences intermittent dinoflagellate blooms with saxitoxin production where invertebrate bioconcentration plays a key role in public health monitoring efforts [31]. In addition, commercial shellfish beds along the Gulf Coast have faced closures due to HAB-associated production of okadaic acid, a source of diarrheic shellfish poisoning (DSP) [32]. Large inland freshwater bodies, such as Lake Erie, are also exhibiting increased frequency and severity of HAB events [33].

#### 2.1.2. Fish

Fish have been indicators of environmental contamination as part of the National Status and Trends Program (NS&T) in the U.S. since 1984 [1]. NS&T includes histopathologic assessments of fish tissues; and these assessments were used to link environmental exposures to the formation of lesions, which often resembled those identified in laboratory animals with similar chemical exposures [1]. Confirmation of similar adverse effects in fish and laboratory animals suggests that similar effects could occur in people sharing the same environments. The first record of a massive fish kill associated with harmful algal bloom formation occurred approximately 1000 years BC in Egypt, and was noted in the bible [34]. HAB-associated fish kills are now commonly recognized, and may serve as rapid indicators of HAB development. HAB-associated fish deaths may occur due to a variety of factors, including direct toxicity of the toxins [15] and hypoxia secondary to high respiration by the algae or increased bacterial respiration during bloom decay [34]. Algae and their products can also cause mechanical or hemolytic damage to gills, ultimately resulting in respiratory failure or fatal metabolic disturbances [34]. Given the complexity of methods by which these events can trigger fish deaths, and the rapid decomposition of fish involved in such events, the pathophysiology of massive, HAB-associated fish kills is often speculative or unknown.

In the late 1990s, investigation of massive fish kills in southeastern U.S. estuaries prompted discovery of an estuarine dinoflagellate, *Pfiesteria piscicida* [35]. Investigators reported human illness following occupational exposure to laboratory cultures of the organism, and fish collected from areas of *P. piscicida* blooms exhibited superficial ulcers [36,37]. Notably, even without evidence that local seafood was contaminated with either *P. piscicida* or any associated toxin, consumers chose not to eat seafood caught from the Chesapeake Bay, or served in restaurants along the shoreline [38]. There is conflicting evidence regarding whether *Pfiesteria piscicida* kills fish by a combination of toxic and physical means or whether the fish kills were due to anoxia or hypoxia with superficial ulcers attributed to *Aphanomyces invadans* infections [39,40,41,42]. Ultimately, no statistical associations were found between exposure to *P. piscicida* cultures or their derivatives and human illness [43,44]. Although the initial concerns about health risks due to *P. piscicida* exposure were not supported by later scientific studies, the attention given to the organism raised public and political awareness about algal bloom events, HABs and their potential ecologic, economic, and health effects.

Recent research demonstrated that microcystins can accumulate in freshwater fish. For example, microcystins were detected in both fresh water samples and fish in aquaculture ponds in Thailand [26]. In the mid-2000s, the Nature Conservancy began restoring the Williamson River Delta Preserve in Oregon, USA with the goal of regenerating habitats essential for the survival of the endangered Lost River sucker (*Deltistes luxatus*), and short-nose sucker (*Chasmistes brevirostris*) [45]. However, lack of recruitment into spawning populations in Upper Klamath Lake is preventing recovery of these typically long-lived species [46]. This disappearance of juvenile suckers coincides with a decrease in water quality, which supported massive cyanobacteria blooms. Bloom decay resulted in high concentrations of cyanotoxins and hypoxic conditions in the Upper Klamath. Short-term microcystin feeding trials, using concentrations approximating those in Upper Klamath Lake, revealed microscopic lesions in exposed suckers similar to those described from other fish species following sub-lethal microcystin exposure [46].

The detrimental impacts of impaired water quality leading to dense cyanobacteria blooms extend well beyond Upper Klamath Lake. Suckerfish are highly valued by the indigenous Klamath Tribe for food and ceremonial purposes [47]. Local cyanobacteria blooms and associated toxins threaten the local ecology, a traditional food source, and ceremonial heritage. Ongoing water and fish monitoring programs can help protect Klamath Tribe members by notifying them when cyanobacteria toxins are accumulating in water and/or fish tissue.

One fish species that has served as a unique local indicator of algal proliferation in freshwater is the pike (*Esox lucius*) [48]. These fish can modify their coloration somewhat to reflect their local environment. During summer 2015, fishermen reported catching “glowing neon fish.” Local fish and wildlife authorities explained that the edges of the pike’s fins, tails, and mouths turn bright neon green as an adaptation for hunting among reeds with admixed algae. While these algae may not produce toxins, the annual appearance of bright green fish signals adaptation to algal proliferation.

Fish are highly susceptible to the toxins produced by another microalga, *Prymnesium parvum* (also known as “golden algae”. *Prymnesium parvum* was first confirmed in west Texas in 1985, and blooms have killed millions of fish; however, it is also likely that the organism was killing fish in the 1950s [49]. Golden algae produce a toxin that asphyxiates gilled animals, including fish, freshwater mollusks, and juvenile frogs. Extended *P. parvum* blooms can drastically affect local ecology with long-term adverse effects on fisheries and economic hardship for those serving recreational anglers [50]. *Prymnesium parvum* was first noticed in Arizona in 2005, possibly carried into the state by migrating water-birds, fishing equipment, water transport, or wind dispersal of dust contaminated with cysts [51]. Although not considered a threat to birds or mammals, golden algae blooms can persist under environmental conditions less favorable to other microalgae, such as cold weather, suggesting that these blooms may be indicators for local or regional ecologic changes.

Because of their high numbers and fixed location, with inability to escape bloom events, farmed fish are especially vulnerable to bloom events and may serve as sensitive bioindicators of HAB formation. In addition, negative economic impacts of HAB events on coastal mariculture can be substantial. . Net-pen liver disease (NLD), is a common disease of farmed Atlantic salmon (*Salmo salar*) from British Columbia and Washington State [52]. Development of severe liver disease appears to be associated with ingestion of microcystin-LR when feeding on natural biota that can proliferate in the nutrient-rich environment of net-pens [53].

Other events associated with toxic algae exposure of fish can be harder to detect, because these events can be small, focal, and ephemeral. Additionally, HAB-exposed fish may be relatively resistant to the toxin’s effects. One example is ciguatera fish poisoning (or ciguatera), a human illness caused by consuming fish that appear to be healthy, but that contain toxins or metabolites produced by the microalga *Gambierdiscus toxicus* [54]. Associated clinical signs in humans include nausea, vomiting, and neurologic symptoms such as tingling fingers or toes. Ciguatera fish poisoning is most commonly associated with ingestion of predatory reef fish harvested from tropical and subtropical waters, such as the Caribbean Sea and the Pacific and Indian Oceans.

#### 2.1.3. Marine Mammals

Marine mammals are long-lived species that tend to live in coastal waters shared by people and feed at the same trophic level as humans. They are susceptible to many recognized human pathogens, including emerging and resurging pathogens such as methicillin-resistant *Staphylococcus aureus*, and *Toxoplasma gondii* [55,56,57]. The health of marine mammals reflects the health of coastal marine waters, including environmental changes triggered by climate change, and presence of chemical contaminants and pathogens. In turn, these animals can serve as “early warning systems” for toxin exposures and potential HAB-associated health effects for people who share the same coastal environments and consume similar foods. HABs are common coastal events, and fossil evidence suggests that repeated mass-strandings of Miocene-era marine mammals in the Atacama Region of Chili may have been HAB-associated [58].

Because many toxins are capable of concentrating and spreading through food webs, one of the most common sources for HAB exposure of marine mammals is their food, including both fish and invertebrates. In this capacity marine mammals often alert resource managers to potential human health risks from consumption of aquatic foods harvested during, or following HAB events. One excellent example is the potent excitatory neurotoxin, domoic acid, which was associated with human amnesic shellfish poisoning Canada in 1987 [59]. In 1998, hundreds of California sea lions (*Zalophus californianus*) died after consuming anchovies (*Engraulis mordax*) that had accumulated high levels of domoic acid after feeding on toxin-producing *Pseudo-nitzschia* spp. [60]. Clinical signs exhibited by domoic acid-exposed sea lions included seizures, head weaving, obtundation, and pruritis. Because no specific antidote exists to treat domoic acid intoxication, affected animals received supportive and symptomatic care, including fluids, diazepam, lorazepam, and phenobarbitone. Despite treatment, over half of the clinically affected animals died, and many animals that were rehabilitated and released re-stranded within four months [61]. Many of the sea lions that survived acute domoic acid intoxication displayed clinical and physical signs of chronic, irreversible brain damage, which was especially apparent in the hippocampus [61].

Many additional California sea lions died from domoic acid poisoning along the California coast during subsequent years [61,62]. In addition to acute death due to neurotoxicity, domoic acid exposure has also been shown to cause cardiac disease [63], abortion, premature parturition, and death of pregnant sea lions [64], thus raising the possibility that similar, non-acute health impacts could also be occurring in humans but are currently under-recognized. Interestingly, the symptomatology and epidemiology of domoic acid toxicosis in California sea lions has evolved over time in response to an apparent increase in toxin-producing blooms [65]. Two distinct clinical syndromes can now be identified; acute domoic acid toxicosis, and chronic epilepsy, reflecting neurologic damage induced by sub-lethal doses of domoic acid. The importance of sub-lethal domoic acid exposure as a cause of epilepsy in humans is unknown.

In addition to consumption of biotoxin-contaminated food and water, both marine mammals and humans can be poisoned via inhalation of aerosolized toxins. In 1996, unprecedented mortality of nearly 150 endangered West Indian manatees (*Trichechus manatus latirostris*) occurred along the southwest Florida coast in association with a bloom of brevetoxin-producing *Gymnodinium breve* (now called *Karenia brevis*) [66]. Affected animals exhibited multi-organ congestion, nasopharyngeal and pulmonary edema and hemorrhage suggestive of brevetoxin poisoning [66], and lymphocytes and macrophages in multiple tissues were immunopositive for brevetoxin. The manatees were thought to have been exposed to brevetoxin through both ingestion and inhalation. Interestingly, weak positive brevetoxin staining was also observed in tissues from control animals, suggesting that manatees are likely repeatedly exposed to brevetoxins [66].

Historically, it was presumed that organisms other than filter-feeding shellfish would not accumulate brevetoxins via the food web. For example, during previous manatee mortality events, brevetoxin exposure was presumed to be via inhalation, as evidenced by pulmonary pathology. However, in 2005, Flewelling *et al.* [17] described two mortality events in which marine mammals were fatally poisoned by brevetoxins during a period when no *K. brevis* bloom was apparent. In 2002, 34 West Indian manatees died in southwest Florida, and in 2004, 107 bottlenose dolphins (*Tursiops truncatus*) died in waters off the Florida panhandle. Although *K. brevis* cell counts were low in seawater samples, brevetoxins were detected in locally-collected fish and seagrass. Exposure was confirmed through biochemical analysis of stomach contents collected during necropsy.

In addition to serving as indicator species for toxic marine HAB events, marine mammals have also been implicated as sentinels for coastal outflows of freshwater HAB toxins. During 2007, 11 dead or dying southern sea otters were recovered from Monterey Bay, California with mild to severe icterus and enlarged, friable livers [21]. Microscopic examination revealed liver lesions consistent with a hepatotoxin, such as mushroom or cyanobacterial poisoning, and biochemical tests confirmed the presence of the potent hepatotoxins, microcystins [21]. Microcystins are typically associated with cyanobacteria found in fresh or estuarine waters, and the findings prompted a search for potential freshwater sources. Pinto Lake, located about 8.5 km inland from where some affected otters were recovered in Monterey Bay, historically had severe *Microcystis* spp. blooms that produced high concentrations of microcystins, and health warnings had been posted near the lake during fall 2007. Miller *et al.* (2010) [21] sampled water and surface algae scum from Pinto Lake through its tributaries to the Pajaro River, which drains directly into Monterey Bay. Microcystin concentrations in Pinto Lake surface scum were extremely high (2.1 million ppb microcystin LA, and 2.9 million ppb total microcystins), and decreased progressively to about 1 ppb in the lower reaches of the Pajaro River. Importantly, both microcystins and the parent cyanobacterium (*Microcystis* spp.) were detected throughout this water system, from Pinto Lake, to within sight of the ocean. Nearly every year since 2007, large and intense *Microcystis* blooms have occurred in Pinto Lake. Although ocean waters and the marine interfaces of coastal rivers flowing into Monterey Bay were biochemically negative for microcystins, samples from the marine outfalls of the Pajaro and Salinas Rivers tested positive for microcystins during the rainy season, when coastal freshwater runoff was highest. A subsequent study has identified microcystin contamination in 15 of 21 freshwater outflows draining into the Monterey Bay National Marine Sanctuary [22]. Nutrient loading was a significant predictor of microcystin concentrations in affected watersheds. In a related study, 100% of assessed coastal fresh water bodies in Southern California tested positive for at least one cyanotoxin, including microcystin, and three sites exceeded California action levels [67].

### 2.2. Birds

In part due to their unique biology, habits and physiology, birds are highly sensitive indicators of local environmental quality. Raptors at the top of the food web, and aquatic birds residing at the land-sea interface often succumb to environmental hazards posed by HABs and their associated toxins. Because birds are numerous and highly visible, avian mass-mortality events often serve as early warning systems for the presence of pesticides and toxins, highlighting local and regional environmental health risks for other animals and people.

For example, the first scientific report of animal mass-mortality due to domoic acid poisoning occurred in brown pelicans (*Pelecanus occidentalis*) and Brandt’s cormorants (*Phalacrocorax penicillatus*) along the central California coast in 1991 [68]. This discovery, along with subsequent mass-mortality of California sea lions due to domoic acid intoxication in 1998, prompted scientists to revisit an avian mass-mortality event in the same region that resulted in deaths of numerous sooty shearwaters (*Puffinus griseus*) in 1961. Large numbers of affected birds acted abnormally, crashed into buildings and regurgitated anchovies throughout the central coast community of Capitola, California. Alfred Hitchcock was a local resident at the time, and researched this event, providing inspiration for his 1963 movie, “The Birds”. Fifty one years later, Bargu *et al.* [69] identified frustules (silica-based exoskeletons) of potentially toxic *Pseudo-nitzschia* spp. in the gastrointestinal tracts of preserved zooplankton collected in 1961 during the same time period and from the same region, strongly supporting the possibility of large-scale avian mortality due to domoic acid intoxication. Numerous additional bird deaths have been associated with domoic acid intoxication in recent years [70,71].

Aquatic birds can also serve as sensitive sentinels for *K. brevis* blooms with brevetoxin production. Kreuder *et al.* [72] investigated sporadic appearance of neurological disease in double-crested cormorants (*P. auritus*) in Florida that may have extended back as much as 30 years. Clinical signs consisted of severe cerebellar ataxia, characterized by a broad-based stance, truncal incoordination, hypermetric gait, and intention tremors. Histopathologic findings were mild and nonspecific, but immunohistochemical staining was positive for brevetoxin. Admittance of cormorants to the Clinic for the Rehabilitation of Wildlife (Sanibel Island, FL, U.S.) with outbreak-specific clinical signs was positively correlated (*p* < 0.05) with concurrent concentrations of *K. brevis* in local water. This cross-correlation coefficient was also significant when increased *K. brevis* levels preceded cormorant admittances by 2, 4, 6, and 8 weeks, suggesting associations between *K. brevis* blooms and local cormorant morbidity.

Interestingly, freshwater and marine-associated toxins can cause avian mortality even when bloom events are not apparent. Avian vacuolar myelinopathy (AVM) is a neurologic disease of birds associated with consumption of submerged vegetation colonized with a toxin-producing cyanobacteria [73,74]. The cyanobacterium *Aetokthonos hydrillicola* has been tentatively identified as the source of this toxin [75]. While the toxin(s) produced have not been fully characterized, these organisms are known to grow abundantly on both introduced *Hydrilla* spp. and on native aquatic plants [76]. Another fascinating aspect of AVM is the ability of these uncharacterized toxins to kill both direct consumers (e.g., American coots, *Fulica americana*), and predatory birds such as bald eagles (*Haliaeetus leucocephalus*) [77]. Coots are exposed through grazing on cyanobacterium-contaminated aquatic vegetation, and affected eagles were shown to develop AVM after consuming affected coots. To date, disease has not been documented in mammals and people, including hunters and others who consume waterfowl [77]. Although there is no direct evidence that humans could be affected, out of an abundance of caution, federal and state agencies have created outreach materials that outline precautions to take when handling and consuming waterfowl that could have vacuolar myelinopathy.

In November–December 2007, a widespread seabird mortality event occurred in Monterey Bay, California (USA) in conjunction with a massive bloom of the dinoflagellate *Akashiwo* *sanguinea* that deposited piles of a foamy substance onshore [78]. Affected birds had water-soaked feathers covered with yellow-green slimy material, and were severely hypothermic. Investigators determined that foam produced by the bloom contained proteins with surfactant properties, which coated the birds’ feathers and neutralized their natural water repellency and insulation [78]. In September 2009, a similar bloom developed off the coast of Washington state, and there was concern about surfers’ exposure to the foam and surfactants generated by *A. sanguinea*. A small study (n = 20) of surfers found that, of 10 respondents who reported no symptoms before surfing, 8 reported nasal congestion or burning post-exposure (Personal communication, Myduc Ta, 19 July 2010). Although this pilot study did not definitively demonstrate that exposure to the chemicals produced by *A. sanguinea* caused specific symptoms in people, the effects observed in birds suggest that humans should avoid exposure to the foams that are the hallmark of this type of bloom.

### 2.3. Domestic Animals

The death of a domestic animal, particularly a pet dog, may be the first indication that a local waterbody has an ongoing toxin-producing HAB event. Dogs are particularly vulnerable because they may swim in waters that smell bad or are visually unappealing to people. They are also likely to lick algae from their fur after they leave the water. Backer *et al.* [79] published a review of canine cyanobacteria poisonings from three different data sources—the Harmful Bloom Related Illness Surveillance System supported by the National Center for Environmental Health, Centers for Disease Control and Prevention; retrospective case files from the Veterinary Medical Teaching Hospital (VMTH), University of California, Davis; and an extensive review of written media reports, scientific and medical manuscripts, and web-based reports of canine cyanobacteria intoxications. Between the 1920s and 2012, 231 unique events and 368 poisonings were reported [79]. Between 2007 and 2011, Departments of Health/Environment from 13 states (California, Florida, Iowa, Kansas, Maryland, Minnesota, Montana, New York, North Carolina, Oregon, Texas, Virginia, and Wisconsin) reported 67 confirmed or suspected cases of HAB-related canine poisonings [79]. Thirty-eight (58%) of the poisonings were fatal. Of the 67 cases, 63 were possibly or confirmed to be associated with exposure to freshwater cyanobacteria HABs. The retrospective review of suspected and confirmed canine cyanobacteria toxin poisoning accessions between 1984 and 2012 from the VMTH necropsy and biopsy cases identified 71 cases that met selection criteria as possible or confirmed cyanotoxin poisoning cases. Of these, 43 dogs (61%) had a moderate to high possibility of microcystin poisoning based on clinical presentation, lesion descriptions, listed differential diagnoses, pathologist comments, and diagnostic test results. Between the late 1920s and mid-2012, historical reports, including media reports, state and federal agency reports, and published literature identified 115 cyanobacteria HAB-related events involving 260 dogs [79]. Two hundred and fifteen (83%) dogs died from the exposures, and 45 (17%) became ill and then recovered.

Stewart *et al.* [5] reviewed reports of cyanobacteria-related poisonings in livestock, wild mammals, and birds. Poisonings have occurred on every continent, and pre-historic mass mortalities of unrelated fauna have been attributed to cyanotoxin poisoning. The early reports of cyanobacteria poisonings included in this review tended to be anecdotal, but included detailed descriptions of bloom events that were temporally and geographically linked with animal deaths. The later reports are less subjective and include clinical and postmortem findings, as well as results from analytic tests developed to detect toxins in clinical specimens. Livestock poisonings are sentinel events indicating contamination of ponds and other water resources that are not routinely monitored. They signal a poisoning risk for other animals using the pond for drinking water and for people using the pond for recreation, including swimming and fishing.

### 2.4. A One Health Approach to HABs

We live in a complex and rapidly changing environment where protection of human health is a growing challenge. Because HAB-associated illness occurs with higher frequency and severity in animals [80], careful investigation and monitoring of animal illnesses and deaths due to biotoxin exposure can expedite recognition and mitigation of potential human health risks. A One Health approach that incorporates animals as key HAB sentinels is needed. The overarching concept of One Health approach includes using exposure and health outcome information learned from animal exposures and outbreaks as components in human risk assessment; applying what we know about human illnesses to animals that might experience similar exposures; developing new public health activities that integrate animal and human data, such as cross-species disease surveillance [80,81]; and creating an environment that facilitates cross-disciplinary collaboration among scientists, veterinarians, medical professionals, and many others [82].

HAB-related animal monitoring coupled with animal and human health event response can be used iteratively to enhance our knowledge of, response to, and prevention of HAB-related exposures and associated illnesses. For example, Amnesic shellfish poisoning from exposure to domoic acid produced by the diatom *Pseudo-nitzschia multiseries* was first documented along the Northeast coast of North America in 1987, when three people died and 100 became ill after eating mussels collected offshore of Prince Edward Island, Canada [59,83]. The first scientific report of domoic acid intoxication of animals or people along the Pacific Coastal North America occurred at about the same time, in 1991 in Monterey Bay, California [70]; although it is possible that earlier west coast events had simply been missed [84]. Additionally associated with blooms of *Pseudo-nitzschia* spp., studies have confirmed bioaccumulation of domoic acid in diverse marine and estuarine invertebrates, characterized by high bioconcentration and slow depuration in some species, notably razor clams [12] mole crabs (*Emerita analoga*) and fat innkeeper worms (*Urechis caupo*) [85]. This was confirmed in work done by Cook *et al.* [86], who reported impaired memory and impaired hippocampal activity in California sea lions (*Alophus califonianus*) exposed to domoic acid. Because these animals rely on spatial memory for foraging and navigation, impaired memory may affect survival in the wild [86]. *Pseudo-nitzschia* blooms of varying intensity with variable domoic acid production are now recognized as an annual event along the Pacific Coast [9], and bloom frequency and severity may be increasing over time [12]. Communication between the human medical community and veterinarians during bloom events could result in both a more proactive response for poisoned marine mammals (e.g., conducting surveillance for strandings) and earlier shellfish bed closings to protect human consumers.

Manatees have also served as sentinels for adverse health outcomes in people. Studies by Bossart *et al.* [66] describing the effects of brevetoxins on wild manatees provided a hypothesis of what human health effects might be expected following brevetoxin exposures. This research indicated that manatees are exposed to brevetoxins by drinking seawater, eating contaminated seagrass, and by breathing contaminated air at the ocean/air interface. These results highlighted the need for a closer look at the effects of both chronic low-level and periodic high-level brevetoxin exposures in people, including lifeguards who are occupationally exposed, and people with underlying respiratory conditions such as asthma. A study by Backer *et al.* [87] assessed Florida lifeguards over time and found that, while the lifeguards reported respiratory symptoms associated with exposure to brevetoxins during Florida red tide events, they did not appear to have adverse effects on respiratory function, as measured by pulmonary function testing [87]. In contrast, Fleming *et al.* [88] reported subtle changes in pulmonary function and increased self-reported respiratory symptoms in people with asthma following exposure to aerosolized brevetoxins. Studies with the same group of asthmatics revealed increases in self-reported symptoms unaccompanied by changes in pulmonary function, even when brevetoxin exposures were low [89]. Linking the manatee epizootic with exposure to brevetoxins revealed a potential gap in information about human exposures. Prior to investigation of the manatee mass-mortality, scientists and beach managers were aware that beach visitors coughed during periods when there was a *K. brevis* bloom event, but potential health consequences were under-recognized. This gap was addressed with scientific studies that revealed not only potential human health effects from these exposures, but also useful clinical information about how asthmatics understand and communicate the severity of their illness [88,89].

Animals may also serve as sentinels for adverse human health effects from freshwater or estuarine cyanobacteria blooms. As noted above, southern sea otters were poisoned by microcystins produced by a freshwater bloom that drained into Monterey Bay [21]. This event, coupled with studies demonstrating microcystin bioaccumulation by *Microcystis*-exposed marine invertebrates could indicate that seafood harvested by people may be unfit to eat due to elevated microcystin concentrations. Also, medical advances in treating microcystin-poisoned animals could help improve medical care for poisoned people. A recent case report documenting successful treatment of confirmed, severe microcystin poisoning in a dog by Rankin *et al.* [90] provided details of the clinical presentation, successful therapy, and subsequent recovery. The dog was exposed to a *Microcystis* spp.-dominated cyanobacterial bloom in a Montana lake. Within hours, the dog was lethargic and anorexic, and clinical signs rapidly progressed to severe depression and vomiting. A complete blood count and serum chemistry panel were indicative of acute hepatic damage, and feces were positive for microcystin-LA on liquid chromatography/mass spectrometry (LCMS). The dog was hospitalized for eight days and treated supportively. Its clinical condition and hematological parameters continued to decline over the first few days of hospitalization. On day 5 of hospitalization, oral treatment with the bile acid sequestrant cholestyramine was inititated, and marked clinical improvement was noted within 48 h. After seventeen days post-exposure, the dog was clinically normal and remained clinically normal at one year post-exposure. Selection of cholestyramine as an inexpensive, easily administered oral medication with the potential to bind microcystin in the digestive tracts of exposed dogs was based on its efficacy in microcystin-exposed rats [91].

Another medication with potential although unproven benefits for treatment of microcystin poisoning in dogs is silibinin. When administered intravenously as Legalon^®^ SIL, this plant derivative has demonstrated efficacy for treatment of Amanita phalloides hepatoxicity [92,93,94]. This flavonolignan appears to interact with specific hepatic transport proteins, blocking cellular amatoxin re-uptake and thus interrupting enterohepatic recirculation [94]. Because microcystin causes hepatic damage through a similar mechanism as amatoxin, administering intravenous silibinin may also limit the effects of microcystin intoxication in exposed animals and people. This possibility appears to be supported by experimental data in microcystin-exposed mice and rats; although livers from rats and mice exposed to microcystin-LR revealed severe hepatocellular necrosis, pre-treatment with silymarin abolished the negative effects of microcystin exposure [92] The successful use of cholestyramine and intravenous silibinin to treat microcystin poisoning in animals provides information to support research on similar medical treatments for people.

Surveillance for HAB-related illnesses in people and animals would enhance our knowledge about the occurrence and distribution of these events. For example, the Centers for Disease Control and Prevention (CDC) supported the Harmful Algal Bloom-related Illness Surveillance System (HABISS) from 2009 until 2013. For the period of 2007–2011, Departments of Health and/or Environment from 11 states funded by the National Center for Environmental Health (NCEH), CDC contributed reports for 4534 HAB events, including environmental data and information on animal and human illnesses [80]. More detailed information describing HAB-related canine poisonings from HABISS and other sources were described by Backer *et al.* [79]. The information recorded in HABISS and efforts to apply these data to support a wide range of state-based public health prevention and response activities indicate that HABs are an environmental One Health issue that needs continuing attention (e.g., [95,96,97]). A new system for surveillance of HAB-related events, disease outbreaks, and cases of illness in people and animals is under development by an internal CDC collaboration between the NCEH and The National Center for Zoonotic, Enteric, and Infectious Diseases. States and other partners will be able to report HAB events, as well as outbreaks and individual cases of HAB-related diseases in people and animals [98].

The number of HAB-related adverse health events affecting animals and people continues to grow. In a one-health approach, information learned about the biological effects from and treatments for exposure to HAB toxins in animals can help determine how to protect and treat humans. Similarly, knowledge gained from the study of human HAB exposures can be used to help protect and treat wild and domestic animals. Table 1 has a summary of the events described in this paper.

## 3. Summary and Conclusions

In the U.S. current state-supported monitoring efforts have prevented many acute foodborne HAB toxin poisonings (*i.e.*, the shellfish poisonings). However, fewer sustainable programs are available to prevent human exposures from HABs in drinking and recreational waters. There are many animals that can serve as sentinels for HAB events, and these sentinels can provide information for public health decision-making. A One Health approach may provide a more complete characterization of HAB events and their consequences, thus limiting both human and animal exposures. Synergized datasets that include simultaneous monitoring of water, algae, people, and animals can more effectively identify high risk areas for HAB events, expedite recognition of potential public health risks, and help catalyze and optimize mitigation and management efforts. Communication and data-sharing across disciplines is critical to our understanding the many nuances of HAB ecology and HAB effects on animals and people.

## Figures and Tables

**Table 1:** Summary of animal sentinel systems and events described in this paper.

Animal Sentinel	Event	References
Aquatic invertebrates and fish		
Marine food web organisms	Brevetoxin bioaccumulation associated with *Karenia brevis* blooms in the Gulf of Mexico and subsequent human poisonings.	Flewelling *et al.* (2005) [17]
Invertebrates	Okadaic acid bioaccumulation in Gulf coast oysters associated with algal blooms.	Gulf Coast oyster reefs may be home to emerging infection threat [32]
Mussels, bivalves, other invertebrates	Environmental contaminant bioaccumulation.	Kimbrough *et al.* (2008) [7]
Marine bivalves, crabs	Domoic acid bioaccumulation in marine bivalves and crabs associated with *Pseudonitzschia* spp. blooms in along western coast of US, from southern California to Alaska, subsequent human and animal (e.g., marine mammals, seabirds) poisonings.	Large bloom of toxic algae underway in Monterey Bay and beyond (2015) Massive domoic acid event in Monterey Bay (2015) [14]
Mussels and clams	Saxitoxin bioaccumulation in mussels and clams, subsequent human paralytic shellfish poisonings.	Lewitis *et al.* (2012) [9]
Mussels	Domoic acid bioaccumulation in mussels, subsequent human amnesic shellfish poisonings.	Perl *et al.* (1960); Bates *et al.* (1998) [59,83]
Invertebrates	Saxitoxin bioaccumulation during dinoflagellate blooms, subsequent poisonings.	Shellfish poisonings [31]
Diverse species, including razor clams (*Siliqua patula*), mole crabs (*Emerita analoga*), fat innkeeper worms (*Urechis caupo*)	Domoic acid bioconcentration and slow depuration during *Pseudonitzschia* spp. blooms.	Trainer and Suddleson (2005) Goldberg (2003) [12,85]
Pike (*Esox Lucius*)	Algal chemical bioaccumulation make the edges of the pike’s fins, tails, and mouths turn bright neon green	The strange case of Yellowknife’s neon green pike [48]
Fish, freshwater mollusks, juvenile frogs	Gill-damaging toxin exposure associated with blooms of *Prymnesium parvum*, subsequent asphyxiation.	Toxic golden alga in Texas [49]
Lost River sucker (*Deltistes luxztus*) and shortnose sucker (*Chasmistes brevirostris*)	Cyanobacteria toxins accumulate in rivers and produce hypoxic conditions, subsequent interference with population recovery.	Burdick and Hewitt (2012); Martin *et al.* (2015); Suckerfish and the Klamath Tribe [45,46,47]
Fish	Cyanobacteria blooms (respiration and bloom decay) produce hypoxic conditions and/or mechanical or hemolytic gill damage and respiratory failure, subsequent fish kills.	Hallegraef (1993) [34]
Commercially-raised marine and estuarine mussels, fish, and other aquatic foods intended for human consumption	Microcystins bioaccumulation associated with cyanobacteria blooms in aquaculture ponds, subsequent poisonings, including net-pen liver disease.	Whangchai *et al.* (2013); Kent *et al*. (1996); Anderson *et al*. (1993) [26,52,53]
Commercially-raised marine and estuarine mussels, fish, and other aquatic foods intended for human consumption	Microcystins bioaccumulation associated with coastal river cyanobacteria blooms contaminating freshwater-to-marine outflows, subsequent animal poisonings.	De Pace *et al.* (2014); Preece *et al.* (2015) [27,28]
Coastal mariculture, including caged yellowtail fish	Toxin bioaccumulation associated with algae blooms.	Hallegraef (1993) [43]
Menhaden and other estuarine fish	Unknown toxin exposure thought to be associated with the presence of *Pfiesteria piscicida*, subsequent fish morbidity and mortality.	Steidinger *et al.* (1996); Glasgow *et al.* (1995); Burkholder *et al.* (1992) [35,36,37]
Marine mammals		
Miocene-era marine mammals	Hypothesized marine HAB-associated toxins bioaccumulation, subsequent mass strandings.	Peyson *et al.* (2013) [58]
Southern sea otters	Microcystins bioaccumulation in oysters, mussels, clams, and snails associated with coastal river cyanobacteria blooms contaminating freshwater-to-marine outflows, subsequent Southern sea otter poisoning.	Miller *et al.* (2010); Lehman *et al.* (2005); Tanner (2005); Gibble *et al.* (2014); Takahashi *et al.* (2014); Wall (2012) [21,22,23,24,25,29]
California sea lions (*Zalophus californianus*)	Domoic acid bioaccumulation associated with a *Pseudo-nitzschia* spp. bloom, subsequent morbidity and mortality of hundreds of sea lions.	Scholin *et al.* (2000); Gulland *et al.* (2002); Bargu *et al.* (2010); Zabka *et al*. (2009); Brodie *et al.* (2006); Goldstein *et al.* (2007) [60,61,62,63,64,65]
West Indian (Florida) manatees (*Trichechus manatus latirostris*)	Brevetoxin bioaccumulation associated with *Karenia brevis* bloom, subsequent morbidity and mortality.	Flewelling *et al.* (2005); Bossart *et al.* (1998) [17,66]
Bottlenose dolphins (*Tursiops truncatus*)	Brevetoxin bioaccumulation associated with *Karenia brevis* bloom, subsequent morbidity and mortality.	Flewelling *et al.* (2005) [17]
Birds		
Brown Pelicans (*Pelecanus occidentalis*) and Brandt’s Cormorants (*Phalacrocorax penicillatus*) in California	Domoic acid bioaccumulation in anchovies during a large *Pseudonitzchia australis* bloom off the California coast, subsequent seabird poisonings.	Work *et al.* (1993); Algae bloom kills sea birds, other sea life in Southern California in record numbers [68,71]
Sooty shearwaters (*Puffinus griseus*)	Domoic acid accumulation in food web, particularly anchovies.	Bargu *et al.* (2012) [69]
Double crested cormorants (*Phalacrocorax auritus*)	Brevetoxin bioaccumulation associated with *Karenia brevis* bloom, subsequent morbidity and mortality.	Kreuder *et al.* (2002) [72]
Bald eagles (*Haliaeetus leucocephalus*), American coots (*Fulica americana*), and other water birds	Cyanobacteria toxin bioaccumulation in association with eating cyanobacterium-contaminated vegetation.	Fischer *et al.* (2006) [77]
Seabirds	Domoic acid bioaccumulation in association with *Pseudonitzchia* spp. blooms, subsequent mortalities.	Fritz *et al.* (1992) [70]
Seabirds	Accumulation of surfactant in coastal waters in association with *Akashiwo sanguinea* bloom, subsequent morbidities and mortalities.	Jessup *et al.* (2009) [78]
Terrestrial animals		
Domestic animals	Cyanobacteria toxins accumulation during cyanobacteria bloom in the Murray River water and subsequent animal poisonings.	Francis (1878) [3]
Domestic and wild animals	Cyanobacteria toxins accumulation in drinking water associated with cyanobacteria blooms, subsequent animal poisonings.	Schwimmer and Schwimmer (1968) [4]
Domestic and wild animals	Cyanobacteria toxins accumulation in river water, subsequent poisonings in animals drinking the water.	Stewart *et al.* (2008) [5]
Dogs	Cyanobacteria toxins accumulation in waterbodies, subsequent poisonings from drinking water, swimming, or licking algae from fur.	Backer *et al.* (2013) [79]
Dog	Microcysin exposure associated with swimming in a blooming lake, subsequent poisoning.	Rankin *et al.* (2013) [90]
